# National Chronic Disease Management Programmes in Irish General Practice-Preparedness and Challenges

**DOI:** 10.3390/jpm12071157

**Published:** 2022-07-17

**Authors:** Meera Tandan, Bebhinn Twomey, Liam Twomey, Mairead Egan, Gerard Bury

**Affiliations:** School of Medicine, University College Dublin, Belfield, D04 V1W8 Dublin, Ireland; bebhinn.twomey@ucdconnect.ie (B.T.); liam.twomey@ucd.ie (L.T.); mairead.egan@ucd.ie (M.E.); gerard.bury@ucd.ie (G.B.)

**Keywords:** chronic disease management, resource profiling, general practice, rural differences, challenges, primary care

## Abstract

Information on the readiness of Irish general practice to participate in structured chronic disease management (CDM) care is limited. This study explores the logistic, staffing, and organizational preparedness of Irish general practice to do so, stratified by their size, location, and training status; implementation challenges were also explored. An anonymous, paper-based random survey was performed. A chi-square test was applied to compare practices by location (urban/rural), post-graduate training status (with/without), and numbers of GMS patient (≥1500/>1500 patients) and prevalence ratio and Poisson regression analysis to examine the relationship of staffing with key variables. Overall, 125/243 practices participated, 22% were rural, 56.6% were post-graduate training practices, and 53.9% had ≥1500 GMS patients. The rural, non-training practices and those with <1500 GMS patients had substantially lower staffing levels. The average number of GPs was significantly less in rural practices; however, the difference was insignificant for nurses. Salary costs for practice nurses in all practices and staff IT training and clinical equipment in smaller practices were important barriers. Most practices reported ‘inadequate’ waiting times for access to almost all referral and paramedical services. The study recommends addressing the staffing, funding, and training challenges within Irish general practice to effectively implement a structured CDM program.

## 1. Introduction

General practice is the first point of contact for individual feeling ill in Irish health care system [1]. Structured general practice programmes for chronic disease management (CDM) have demonstrated benefits such as improved monitoring, better compliance and some improved outcomes [2,3,4,5,6]. Ireland’s Department of Health has identified structured care of chronic diseases in general practice as a core component of future health policy; in 2020 the Health Service Executive (HSE) established a CDM care system for general practice [7,8].

Approximately 3500 GPs provide care to Ireland’s 5 million people; low income and younger/older age thresholds provide ‘free at the point of delivery’ care to around one-third of the population through the state’s General Medical Services (GMS) Scheme; around 29 million consultations occur annually in Irish general practice [9]. The HSE says that over 85% of GPs have joined the CDM programme in its initial year [10].

Specific challenges and deficiencies have also been identified in general practice in the management of chronic diseases, related to resources, staffing, access, case finding and other issues [11,12,13,14,15,16]. In the UK, Anwar et al. noted that general practices in deprived areas had significantly higher burdens of chronic diseases, with access to care in busy practices becoming increasingly difficult [17]. Pericin et al. identified the inadequacy of coding of chronic diseases in Irish general practices as a potential barrier to implementation of structured care [18].

Ireland’s CDM structured programme is targeted at patients with Type 2 diabetes, asthma, chronic obstructive pulmonary disease (COPD) or cardiovascular disease (including heart failure, coronary artery disease, stroke, and atrial fibrillation). Its components include opportunistic case finding, structured CDM treatment and annual reviews. Practice nursing roles are highlighted in the CDM programme and have been shown to impact positively on patient experience of care, although experience elsewhere has identified the significant challenges facing general practice in building nursing capacity for this purpose [19,20].

Limited data is available to indicate the preparedness of Irish general practice to take on these roles, the views of GPs about implementation issues or whether differences in preparedness exist in different practice settings. This study set out to describe the logistic, staffing, and organisational preparedness of Irish general practice and to explore the challenges reported by participating GPs.

## 2. Methods

This is a cross-sectional study conducted among general practices within the network of University College Dublin (UCD) Academic General Practice located across Ireland. These practices serve as the first point of contact for a patient for any cause, including chronic conditions.

An anonymous, paper-based survey questionnaire was used to collect data. The survey questionnaire was divided into seven main sections with five to six variables within each section. The first section (practice human resources and infrastructure) included the number of full-time/part-time GPs, nurses, practice managers and other health care and administrative staff and number of GP and nurse consulting rooms. The second section (CDM resources) comprised current number of doctors/nurses involved in HSE CDM program and adequacy of physical infrastructure to implement CDM, quality of practice equipment along with adequacy of CDM training resources to staff. The third section (staff availability and training) examined readiness to implement HSE CDM for specific diseases such as Asthma, COPD, IHD and Diabetes. The fourth section (availability and annual calibration of equipment required for CDM)—12 lead ECG machine, ABP monitors, glucometer, weighing scales, peak flow meter and spirometry. The fifth section (access to and adequacy of waiting times of nearby services) examined cardiac echo, exercise stress test, physiotherapy, dietitian, retinal screening, and smoking cessation. The sixth section (views on potential barriers to HSE CDM program implementation) this examined cost and sourcing of additional staff, staff training both in IT system and clinical equipment use, GDPR concerns, IT security concerns and practice building infrastructure. The final section examined views on the expansion of the CDM programme to other issues such as mental health, traveller health, obesity, osteoporosis. In addition, variables such as practice location (rural/non-rural), number of GMS patients served, and post-graduate training practice (yes/no) were also explored. The selection of variables in the study was based on both a literature search and research team consultation with senior GPs working within Irish general practices.

The study questionnaire was piloted and refined prior to circulation. The questionnaire was circulated to a random sample of 243 general practices, drawn from a network of almost 700 practices associated with UCD Academic General Practice. In case of non-response to the first request practices received two reminders about the study. Research ethics approval was granted by the UCD Human Research Ethics Committee (Ethic approval number—UTMREC-SM-E-PG-20-82-Twomey-Bury).

Issues such as rural setting, post-graduate training status and numbers of GMS patients (≤1500/>1500 patients) have previously been proposed as influences on the activities of Irish general practices and therefore constitute a focus for the analysis of data in this study [21]. The differences for these outcome variables were calculated for staffing levels, facilities, and identification of barriers to implementation.

Chi-square tests of independence were applied to see the differences between groups for categorical data and Wilcoxon rank sum test to numeric data; logistic regression examined the relationship between medical staffing (categorical variable) and Poisson regression analysis for total staff (count data) with key variables. A *p*-value of <0.05 was considered significant. All the analysis was performed in R version 4.0.3 (10 October 2020). The outcomes of the study are presented in tables and figures where appropriate. Denominators vary, depending on responses to questions.

## 3. Results

**Figure 1:**
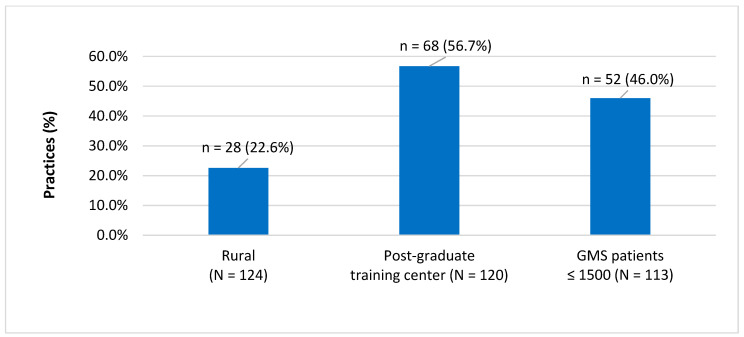
Characteristics of practice by location, training, and GMS patients’ size.

In all, 125/243 (51.4%) practices responded to the survey. Figure 1 summarises the practices locations, training roles and size. Overall, 28/124 (22%) practices are in (self-identified) rural areas, 68/120 (56.6%) are post-graduate training practices and 61/113 (53.9%) have more than 1500 GMS patients attending the practice.

(Table 1), of all practices, 123/125 (98.4%) reported involvement in the CDM programme; of these 122/123 (99.2%) reported doctor and 119/123 (96.7%) nurse involvement in the CDM program. Nearly 80% of the practices had two or more GPs and 66% had two or more nurses involved in the CDM program. It is striking that rural practices, smaller practices and non-training practices have significantly fewer doctors although practice nurse numbers are not significantly different in rural/non-rural, with/without training practice, but the number of more than two nurses’ involvement in the CDM program was significantly higher in larger practices (≥1500 GMS patients).

The mean number of total staff in the practice was 10.2, with 3.2 GPs and 2.0 practice nurses (Table S1). The rural, non-training practices and those with less than 1500 GMS patients had substantially lower staffing levels. The average number of GPs was significantly less in rural practices; however, the difference was not significant for nurses except for training practice and practice with more than 1500 GMS patients (Figure S1).

Table 2 shows that for comparable data items, these results are similar to those of a large recent workload study in Irish general practice [9]. Post-graduate training practices are probably over-represented among responders, at 56.6%; around 40% of Irish general practices are likely to be involved in post-graduate training.

Prevalence ratio estimation showed significantly less involvement of more than two doctors in the CDM programme in rural practices (PR 0.24, CI 0.07–0.80). In comparison, the involvement of more than two doctors was more likely in practices that are post-graduate training centres (PR 2.32, CI 1.20–4.46) and that had more than 1500 GMS patients (PR 2.16, CI 1.14–4.11). Likewise, the involvement of more than two nurses in the CDM programme was more likely to be in large practices (≥1500 GMS patients) compared to small practices (PR 9.41, CI 2.54–34.90) when considered practice location and post-graduate training in the model (Table 3).

Almost all practices have the equipment needed to implement the CDM programme; spirometry is not essential equipment but is available in a significant proportion of practices. (Table 4). More than 90% of the practices routinely code for asthma, COPD, heart failure and diabetes, using their IT systems.

**Figure 2:**
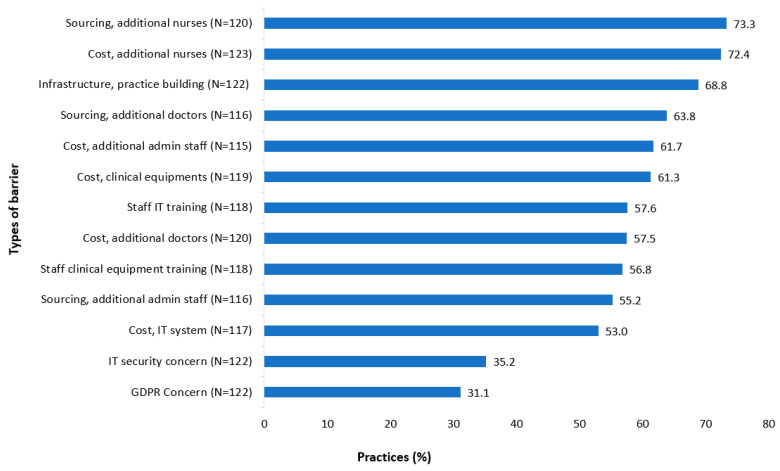
Barriers to implementing HSE CDM programme in general practice.

When asked about possible barriers to implementing the CDM programme, respondents identified a range of issues summarised in Figure 2. Recruitment (73.3% agree) and salary costs (72.4% agree) for practice nurses and inadequate premises (68.8% agree) were among the key barriers identified. Of note, few differences in these issues emerged between rural/non-rural practices, training/non-training practices and large/smaller practices. However, staff training on IT and clinical equipment were significantly more likely to be identified as barriers in small practices and non-training practices (Table S2).

**Figure 3:**
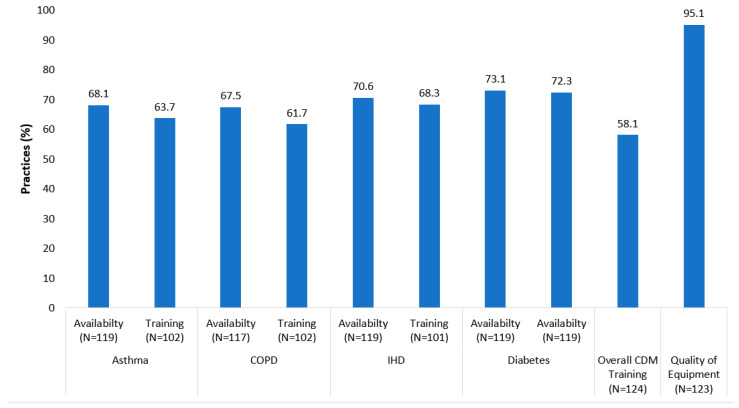
Staff availability and training adequacy to implement different chronic conditions under HSE CDM programme in general practice. COPD = Chronic Obstructive Pulmonary Disease, IHD = Ischemic Heart Disease.

Between 27–30% of the practices report that they had inadequate numbers of staff to implement the HSE CDM program (Figure 3). Similarly, CDM-related training was reported as inadequate in 42% of the practices (35% had inadequate training related to COPD and asthma, 31% IHD, and 28% diabetes). These issues did not differ by practice location, size or training status (Table S3).

**Figure 4:**
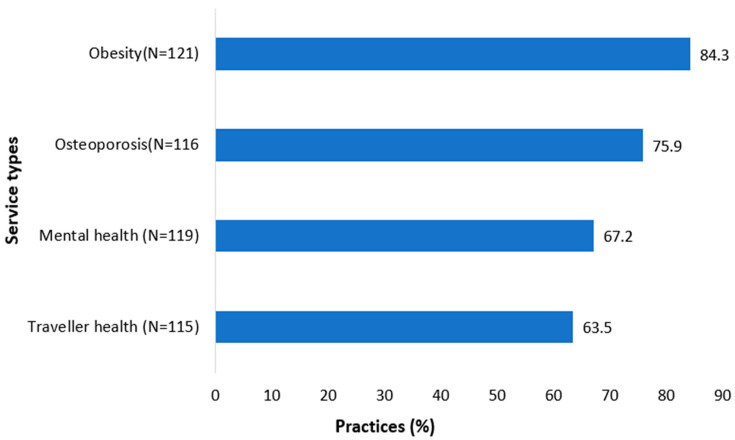
Agreement on the expansion of other health services to HSE CDM programme in general practice.

The majority of the practices agreed that the current HSE CDM programme should be expanded to include other issues (85% agreed for obesity expansion, 76% for osteoporosis, 67% for mental health, and 63% for traveller health) (Figure 4). Again, these issues did not differ by location, size or training status (Table S3).

Almost all practices have access to physiotherapy and retinal screening and smoking cessation services. In contrast, 25 to 31% of the practices did not have access to exercise stress tests and cardiac echo. However, most practices report ‘inadequate’ waiting times for almost all paramedical services (Table S4).

## 4. Discussion

The study data show significant engagement by Irish general practices with the HSE CDM programme. However, the availability of the staff and other resources needed to implement the programme varies greatly. Our data demonstrate that smaller practices, non-training practices and those in rural areas are less likely to be adequately staffed with GPs or practice nurses. These findings are similar to those reported from other European countries and the United States where primary care practices more often lack physician and nurse practitioners in rural areas compared to urban ones [22,23]. This indicates that closing rural-urban gaps in access to primary care clinicians and other resources requires intensive efforts targeting rural areas. However, the important strengths identified in our study include most practices having several GPs involved, general involvement of practice nursing staff and extensive electronic coding of relevant illnesses.

When questioned about barriers to effective implementation, training and staffing issues arise in 27% to 42% of all practices. In particular, the challenges of recruiting and funding practice nursing posts was highlighted. A national-level survey conducted back in 2011 also stressed increased staff workload and lack of appropriate funding as the critical barrier to CDM implementation in Irish general practices [11]. Similar issues has been highlighted in the international literature [18,19] and our group has previously reported on the importance of expanding practice nursing for Irish general practice [24].

The ICGP 2022 submission to the Oireachtas Health Committee (i) highlights the challenges of inadequate staffing and workload pressures on general practice nationally; in particular, it points out the potential unsustainability of one/two doctor practices. Our paper provides supporting evidence for this position in terms of overall staffing concerns and goes further by indicating specific groups of practices which are most at risk. Our study shows that single-handed GP practices are twice as likely to be in rural areas (48% versus 22.5% overall), to be non-training practices (65.2% versus 43.6% overall) and to have GMS lists of less than 1500 patients (85% versus 46.1%), thus attracting less health service supporting funds. The challenges of the workload associated with the CDM programmes will add considerably to pressures on such practices. If rural areas cannot be served by viable general practices providing the full range of services, then local communities will suffer and the ‘knock-on’ effects on other parts of the health service may become an issue. Because single-handed practices offer comparable quality of care to large practices but tend to refer more patients with chronic disease to secondary care as they do not have opportunity to exercise sharing task, knowledge and skills within their own practices [25].

The training needs identified by respondents are a further implementation issue to be addressed.

It is striking that most respondents expressed their views on the potential to further develop the CDM programmes beyond the current group of chronic diseases. Significant majorities were in favour of each new programme proposed with more than two-thirds supporting obesity, mental health and osteoporosis programmes. This strong support for expansion should inform future health service planning.

Limitations of the study include its origins in an academic pool of GPs, a limited response rate and an over-representation of post-graduate training practices. However, random selection of GPs from the very large academic pool may mitigate possible selection bias; it is also noteworthy that a response rate of over 50% was achieved when practices were under very significant pressures because of the COVID-19 pandemic.

## 5. Conclusions

Our study highlights the staffing, funding, and training challenges to be addressed by Irish general practice in implementing CDM programmes, in spite of the clear enthusiasm to participate in structured CDM programmes. Small and rural practices may be particularly stretched in delivering such additional services.

## Figures and Tables

**Table 1:** Practice staff (GP and nurses) involvement in the CDM by practice location, size, and post-graduate training roles.

Characteristics	Rural				Post Graduate Training Centre				GMS Patients			
GPs	All (*N* = 122) (*n*, %)	Yes (*N* = 28) (*n*, %)	No (*N* = 94) (*n*, %)	*p* Value	All (*N* = 118) (*n*, %)	Yes (*N* = 68) (*n*, %)	No (*N* = 50) (*n*, %)	*p* Value	All (*N* = 111) (*n*, %)	≤1500 (*N* = 50) (*n*, %)	>1500 (*N* = 61) (*n*, %)	*p* Value
1 GP (doctor)	25 (20.5)	12 (42.8)	13 (13.8)	0.01	23 (19.5)	8 (11.8)	15 (30.0)	<0.05 *	20 (18.0)	17 (34.0)	3 (4.9)	0.001
2–3 GPs	70 (57.4)	12 (42.8)	58 (61.7)		69 (58.5)	41 (60.3)	28 (56.0)		64 (57.7)	31 (62.0)	33 (54.2)	
≥4 GPs	27 (22.1)	4 (14.4)	23 (24.5)		26 (22.0)	19 (27.9)	7 (14.0)		27 (24.3)	2 (4.0)	25 (40.9)	
**Practice nurses**	**All (*N* = 119)** **(*n*, %)**	**Yes** **(N = 27)** **(*n*, %)**	**No** **(*N* = 92)** **(*n*, %)**	***p* Value**	**All (*N* = 115)** **(*n*, %)**	**Yes (*N* = 67)** **(*n*, %)**	**No** **(*N* = 48)** **(*n*, %)**	***p* Value**	**All (*N* = 108)** **(*n*, %)**	**≤1500** **(*N* = 47)** **(*n*, %)**	**>1500** **(*N* = 61)** **(*n*, %)**	***p* Value**
1 nurse	53 (44.5)	10 (37.0)	43 (46.7)	0.63	52 (45.2)	27 (40.3)	25 (52.1)	0.12	48 (44.4)	29 (61.7)	19 (31.1)	0.001
2–3 nurses	56 (47.1)	14 (51.9)	42 (45.7)		54 (46.9)	32 (47.8)	22 (45.8)		50 (46.3)	18 (38.3)	32 (52.5)	
≥4 nurses	10 (8.4)	3 (11.1)	7 (7.6)		9 (7.9)	8 (11.9)	1 (2.1)		10 (9.3)	0 (0.0)	10 (16.4)	

**Table 2:** Study data (*n* = 125) compared to Collins et al. [9]. (*n* = 507).

	Mean Number of GPs	At Least One Practice Nurse	Rural Location	Single GP
Our study	3.2	95%	22%	20.5%
Collins et al. [9]	2.9	94%	18.9%	18%

**Table 3:** Model showing association between staff involvement in CDM programme by practice location, size, and post-graduate training.

	Total Staff ¶				More Than 2 Doctors Involve in CDM £				More Than 2 Nurse Involve in CDM £			
	Model 1 OR [CI]	Model 2 OR [CI]	Model 3 OR [CI]	Model 4 OR [CI]	Model 1 PR [CI]	Model 2 PR [CI]	Model 3 PR [CI]	Model 4 PR [CI]	Model 1 PR [CI]	Model 2 PR [CI]	Model 3 PR [CI]	Model 4 PR [CI]
**Rural**	1.05 [0.89–1.23]	0.79 [0.68–0.92] *		1.01 [0.86–1.19]	0.34 [0.12–0.97] *	0.19 [0.06–0.60] *		0.24 [0.07–0.80] *	1.91 [0.55–6.21]	0.82 [0.29–2.23]		1.85 [0.51–6.64]
**GMS > 1500 patients**	1.92 [1.68–2.20] *		1.80 [1.58–2.06] *	1.81 [1.57–2.08] *	2.12 [1.25–3.61] *		3.08 [1.59–4.14] *	2.32 [1.20–4.46] *	9.47 [2.66–33.78] *		7.71 [2.32–25.51] *	9.41 [2.54–34.90] *
**Postgraduate training**		1.43 [1.26–1.62] *	1.24 [1.09–1.41] *	1.24 [1.09–1.41] *		1.92 [1.20–3.06] *	2.60 [1.08–9.32] *	2.16 [1.14–4.11] *		3.65 [1.14–6.14] *	2.17 [0.78–6.02]	2.14 [0.76–6.02]

* Significant at *p* value < 0.05, ¶Poisson regression analysis, £ Prevalence ratio. Shadow in the table refers that the variable is not included in the model, example in the model 1, shadow in last row first column means postgraduate training is not included in model 1.

**Table 4:** Equipment and disease coding within practices.

Equipment	Rural				Post-Graduate Training				GMS Patients			
	All	Yes	No	*p* Value	All	Yes	No	*p* Value	All	≤ 1500	≥ 1500	*p* Value
12 lead ECG machine (*N* = 123)	113 (91.8)	28 (24.7)	85 (75.3)	0.11	110 (89.4)	66 (60.0)	44 (40.0)	<0.05	102 (82.9)	45 (44.1)	57 (55.9)	0.18
ABP monitor (*N* = 123)	122 (99.2)	28 (22.9)	94 (77.1)	0.93	119 (96.7)	68 (57.1)	51 (42.8)	NA	111 (90.2)	51 (45.9)	60 (54.0)	0.46
Sphygmomanometer (*N* = 123)	121 (98.4)	28 (23.1)	93 (76.8)	0.92	117 (95.1)	67 (57.3)	50 (42.7)	0.92	110 (89.4)	51 (46.4)	59 (53.6)	0.93
Glucometer (*N* = 123)	123 (100.0)	28 (22.7)	95 (77.3)	NA	119 (96.7)	68 (57.1)	51 (42.9)	NA	112 (91.0)	52 (46.4)	60 (53.6)	NA
Weighing scales (*N* = 123)	123 (100.0)	28 (22.7)	95 (77.3)	NA	119 (96.7)	68 (57.1)	51 (42.9)	NA	112 (91.0)	52 (46.4)	60 (53.6)	NA
Height measurement (*N* = 123)	122 (99.2)	28 (22.9)	94 (77.1)	0.96	118 (95.9)	67 (56.8)	51 (43.2)	0.92	111 (90.2)	52 (46.8)	59 (53.1)	0.94
Peak Flow Meter (*N* = 122)	113 (92.6)	28 (24.8)	85 (75.2)	0.12	110 (90.2)	63 (57.3)	47 (42.7)	0.75	102 (83.6)	46 (45.1)	56 (54.9)	0.73
Spirometry (*N* = 120)	57 (47.5)	13 (22.8)	44 (77.2)	0.98	54 (45.0)	36 (66.7)	18 (33.3)	0.07	50 (41.7)	16 (32.0)	34 (68.0)	<0.05
**Practice routinely** **codes for:**												
Asthma (*N* = 122)	110 (90.2)	26 (23.6)	84 (76.4)	0.73	108 (88.5)	66 (61.1)	42 (38.9)	<0.05	101 (82.8)	46 (45.5)	55 (54.4)	0.51
COPD (*N* = 121)	111 (91.7)	27 (24.3)	84 (75.7)	0.45	108 (89.3)	64 (59.3)	44 (40.7)	0.16	102 (84.3)	48 (47.1)	54 (52.9)	0.97
Heart disease (*N* = 122)	111 (91.0)	27 (24.3)	84 (75.7)	0.45	108 (88.5)	64 (59.3)	44 (40.7)	0.32	101 (82.8)	48 (47.5)	53 (52.5)	0.75
Diabetes (*N* = 122)	114 (93.4)	27 (23.7)	87 (76.3)	0.68	111 (91.0)	66 (59.5)	45 (40.5)	0.13	104 (85.2)	49 (47.1)	55 (52.9)	0.95

NA = Chi-square test not applicable.

## Supplementary Materials

The following supporting information can be downloaded at: https://www.mdpi.com/article/10.3390/jpm12071157/s1. Figure S1: Overall staffing by practice location, size, and post-graduate training; Table S1: Overall staffing by practice location, size, and post-graduate training; Table S2: Perceived barriers to implement HSE CDM programme by practice location, size, and post-graduate training; Table S3: CDM resources, and expansion of other services by practice lo-cation, size, and post-graduate training; Table S4: Nearby local services and satisfaction towards waiting times by practice location, size, and post-graduate training.
