# Supplementary material for: National Chronic Disease Management Programmes in Irish General Practice-Preparedness and Challenges
Source: J Pers Med. 2022 Jul 17;12(7):1157. doi: 10.3390/jpm12071157 (PMC9323818; doi:10.3390/jpm12071157)
Supplement: Supplementary file 1 [file jpm-12-01157-s001.zip › jpm-1747773-supplementary.pdf]

Supplementary Materials

Supplementary table S1: Overall staffing by practice location, size, and post-graduate training.

Characteristics	All (N=125) (Mean, SD, R)	Rural (N=124)		P value	Post-graduate training centre (N=120)		P value	GMS patients (N=113)		P value
		Yes (N=28) (Mean, SD, R)	No (N=96) (Mean, SD, R)		Yes (N=68) (Mean, SD, R)	No (N=52) (Mean, SD, R)		<=1500 (N=52) (Mean, SD, R)	>1500 (N=61) (Mean, SD, R)	
Full-time GP	2.1, 1.9, 0-18	1.7, 1.8, 0-7	2.2,1.9, 0-18	<0.05	2.2,1.4, 0-7	1.9, 2.4, 0-18	<0.05	1.2,0.7,0-3	2.6,1.4, 0-7	0.001
Part time GP	1.1, 1.3, 0-6	0.6, 0.9, 0-4	1.3,1.4, 0-6	<0.05	1.1, 1.2, 0-5	1.2, 1.4, 0-6	0.74	0.7,1.1,0-4	1.3, 1.4, 0-6	<0.05
**Total GP**	**3.2, 2.3, 1-20**	**2.4, 1.9, 1-8**	**3.4, 2.4, 1-20**	**0.001**	**3.3, 1.7, 1-8**	**3.1, 2.8, 01-20**	**0.19**	**2.0,0.9, 1-5**	**4.0,1.8,1-8**	0.001
Full-time GP Registrars	0.5, 0.6, 0-2	0.5, 0.7, 0-2	0.4, 0.6, 0-2	0.69	0.8, 0.6, 0-2	0.0,0.0, 0-0	0.001	0.3,0.5,0-1	0.7,0.6,0-2	0.001
Part-time GP Registrars	0.04, 0.2, 0-1	0.07, 0.3, 0-1	0.03, 0.2, 0-1	0.35	0.06, 0.2, 0-1	0.02, 0.1, 0-1	0.29	0.04,0.2,0-1	0.03,0.2,0-1	0.88
**Total GP Registrars**	**0.5, 0.6, 0-2**	**0.6, 0.7, 0-2**	**0.4, 0.6, 0-2**	**0.45**	**0.9, 0.5, 0-2**	**0.02, 0.1, 0-1**	**0.001**	0.3,0.5,0-1	0.7,0.6,0-2	0.001
Full-time Practice Nurses	0.9, 1.2, 0-6	1.1, 1.4, 0-6	0.9,1.2, 0-6	0.68	1.1,1.4, 0-6	0.7, 0.9, 0-4	0.10	0.5,0.6, 0-2	1.4, 1.5, 0-6	0.001
Part-time Practice Nurses	1.02, 1.1, 0-5	1.1, 1.3, 0-5	1.0, 1.1,0-5	0.92	1.06, 1.2, 0-5	0.9, 1.1, 0-5	0.74	0.9,0.8, 0-2	1.2,1.4, 0-5	0.80
**Total Practice Nurses**	**2.0, 1.2, 0-6**	**2.1, 1.2, 0-6**	**1.9,1.1,0-6**	**0.54**	**2.2, 1.2, 1-6**	**1.7, 0.9, 0-5**	<0.05	1.4,0.6, 0-2	2.5, 1.3, 1-6	0.001
Full-time Practice Managers	0.6, 0.5,0-2,	0.4, 0.5, 0-1	0.7, 0.5, 0-2	0.01	0.7, 0.5, 0-2	0.5, 0.5, 0-1	<0.05	0.4,0.5,0-1	0.7,0.5, 0-2	0.001
Part-time Practice Managers	0.3, 0.5, 0-3	0.2, 0.4, 0-1	0.3, 0.6, 0-3	0.99	0.3, 0.5, 0-2	0.2, 0.4, 0-2	0.27	0.2,0.4, 0-1	0.3,0.5,0-2	0.77
**Total Practice Managers**	**0.9, 0.6, 0-4**	**0.6, 0.5, 0-2**	**0.9, 0.5, 0-4**	**0.0**1	**1.03, 0.4, 0-2**	**0.7, 0.5, 0-2**	**0.001**	0.6,0.5, 0-1	1.07, 0.4, 0-2	0.001
**Total other admin staff**	3.4, 2.5, 0-15	3.2, 3.3, 0-15	3.5, 2.3,0-14	<0.05	3.8, 2.6, 0-15	2.9, 2.1, 0-11	0.01	2.3,1.3,0-6	4.5,3.0,0-15	0.001
**Total staff**	**10.2, 5.7, 2-32**	**9.2, 6.9, 3-32**	**10.5, 5.3, 0-28**	<0.05	**11.3, 5.5, 3-32**	**8.7, 5.3, 2-27**	**0.001**	6.7,2.6,2-13	13.0,6, 6-32	0.001

P-values calculated using Wilcoxon rank sum test

Supplementary table S2: Perceived barriers to implement HSE CDM programme by practice location, size, and post-graduate training.

Characteristics	Rural				Postgraduate training				GMS patients			
	All	Yes	No	P value	All	Yes	No	P value	All	<=1500	>1500	P value
**Cost of additional; Doctors**	N=120	N=26	N=94		N=116	N=64	N=50		N=110	N=50	N=60	
n (%)	69 (57.5)	17 (65.4)	52 (55.3)	0.49	64 (55.2)	34 (51.5)	30 (60.0)	0.47	62 (56.4)	29 (58.0)	33 (55.0)	0.90
**Cost of additional; Practice Nurse**	N=123	N=28	N=95		N=119	N=68	N=51		N=112	N=52	N=60	
n (%)	89 (72.4)	21 (75.0)	68 (71.6)	0.91	84 (70.6)	49 (72.1)	35 (68.6)	0.84	79 (70.5)	37 (71.2)	42 (70.0)	0.97
**Cost of additional; Admin staff**	N=115	N=27	N=87		N=111	N=86	N=45		N=106	N=49	N=57	
n (%)	71 (61.7)	19 (59.0)	52 (59.1)	0.41	66 (59.5)	40 (60.6)	26 (57.8)	0.92	63 (59.4)	32 (65.3)	31 (54.4)	0.34
**Sourcing of additional; Doctors**	N=116	N=26	N=90		N=112	N=63	N=49		N=107	N=49	N=58	
n (%)	74 (63.8)	19 (73.1)	55 (61.1)	0.37	70 (62.5)	38 (60.3)	32 (65.3)	0.73	67 (62.6)	32 (65.3)	35 (60.3)	0.74
**Sourcing of additional; Practice Nurses**	N=120	N=27	N=93		N=116	N=67	N=49		N=109	N=50	N=59	
n (%)	88 (73.3)	22 (81.5)	66 (71.0)	0.40	84 (72.4)	50 (74.6)	34 (69.4)	0.68	79 (72.5)	39 (78.0)	40 (67.8)	0.33
**Sourcing of additional; Admin staff**	N=116	N=26	N=90		N=112	N=65	N=47		N=106	N=47	N=59	
n (%)	64 (55.2)	18 (69.2)	46 (51.1)	0.16	60 (53.6)	34 (52.3)	26 (55.3)	0.90	57 (53.8)	30 (63.8)	27 (45.8)	0.09
**Cost of IT system**	N=117	N=28	N=89		N=113	N=66	N=47		N=106	N=49	N=57	
n (%)	62 (53.0)	16 (57.1)	46 (51.7)	0.77	57 (50.4)	34 (51.5)	23 (48.9)	0.94	54 (50.9)	23 (46.9)	31 (54.4)	0.57
**Cost of clinical equipment's**	N=119)	N=28	N=81		N=115	N=67	N=48		N=108	N=49	N=59	
n (%)	73 (61.3)	20 (71.4)	53 (58.2)	0.30	69 (60.0)	38 (56.7)	31 (64.6)	0.51	64 (59.2)	32 (65.3)	32 (54.2)	0.33
**Staff IT training**	N=118	N=28	N=90		N=114	N=63	N=51		N=108	N=51	N=57	
n (%)	68 (57.6)	15 (53.6)	53 (58.9)	0.78	65 (57.1)	30 (47.6)	35 (68.6)	<0.05	60 (55.6)	35 (68.6)	25 (43.9)	<0.05
**Staff clinical equipment training**	N=118	N=28	N=90		N=114	N=63	N=51		N=108	N=50	N=58	
n (%)	67 (56.8)	15 (53.6)	52 (57.8)	0.86	64 (56.1)	29 (46.0)	35 (68.6)	<0.05	59 (54.6)	33 (66.0)	26 (44.8)	<0.05
**GDPR concern**	N=122	N=28	N=94		N=118	N=68	N=50		N=111	N=52	N=59	
n (%)	38 (31.1)	10 (35.7)	28 (29.8)	0.72	35 (29.7)	21 (30.9)	14 (28.0)	0.89	32(28.8)	17 (32.7)	15 (25.4)	0.53
**IT security concern**	N=122	N=28	N=94		N=118	N=68	N=50		N=111	N=52	N=59	
n (%)	43 (35.2)	12 (42.9)	31 (32.9)	0.46	41 (34.7)	23 (33.8)	18 (36.0)	0.96	37(33.3)	20 (38.5)	17 (28.8)	0.38
**Practice building infrastructure**	N=122	N=27	N=95		N=119	N=68	N=51		N=111	N=52	N=59	
n (%)	84 (68.8)	19 (70.4)	65 (68.4)	0.99	80 (67.2)	47 (69.1)	33 (64.7)	0.76	75(67.6)	36 (69.2)	39 (66.1)	0.88

P-values calculated using chi-square test

Supplementary table S3: CDM resources, and expansion of other services by practice location, size, and post-graduate training.

Characteristics	Rural				Post graduate training centre				GMS patients			
	All	Yes	No	P value	All	Yes	No	P value	All	<=1500	>1500	P-value
**CDM resources overall**												
CDM training resources available to staff	***(N=124)***	***(N=28)***	***(N=96)***		***(N=120)***	***(N=68)***	***(N=52)***		***(N=113)***	***(N=52)***	***(N=61)***	
Adequate (n, %)	72 (58.1)	15 (53.6)	57 (59.4)	0.74	69 (57.5)	40 (58.8)	29 (55.8)	0.88	60 (53.1)	21 (18.6)	39 (34.5)	<0.05
Inadequate (n, %)	52 (41.9)	13 (46.4)	39 (40.6)		51 (42.5)	28 (41.2)	23 (44.2)		53 (46.9)	31 (27.4)	22 (19.5)	
Physical infrastructure to effectively participate in the CDM Programme	***(N=124)***	***(N=28)***	***(N=96)***		***(N=120)***	***(N=68)***	***(N=52)***		***(N=113)***	***(N=52)***	***(N=61)***	
Adequate (n, %)	101 (81.5)	23 (82.1)	78 (81.2)	0.99	97 (80.8)	59 (86.8)	38 (73.1)	0.09	91 (80.5)	41 (78.9)	50 (82.0)	0.86
Inadequate (n, %)	23 (18.5)	5 (17.9)	18 (18.8)		23 (19.2)	9 (13.2)	14 (26.9)		22 (19.5)	11 (21.1)	11 (18.0)	
Quality of practice equipment used in CDM	***(N=123)***	***(N=28)***	***(N=95)***		***(N=119)***	***(N=68)***	***(N=51)***		***(N=112)***	***(N=51)***	***(N=61)***	
Adequate (n, %)	117 (95.1)	27 (96.4)	90 (94.7)	0.99	113 (95.0)	65 (95.6)	48 (94.1)	0.92	106 (94.6)	49 (96.1)	57 (93.4)	0.69
Inadequate (n, %)	6 (4.9)	1 (3.6)	5 (5.3)		6 (5.0)	3 (4.4)	3 (5.9)		6 (5.4)	2 (3.9)	4 (6.6)	
**CDM resource for Asthma**												
CDM staff availability	***(N=119)***	***(N=28)***	***(N=91)***		***(N=115)***	***(N=67)***	***(N=48)***		***(N=108)***	***(N=49)***	***(N=59)***	
Adequate (n, %)	81 (68.1)	22 (78.6)	59 (64.8)	0.26	77 (67.0)	48 (71.6)	29 (60.4)	0.29	74 (68.5)	33 (67.3)	41 (69.5)	0.97
Inadequate (n, %)	38 (31.9)	6 (21.4)	32 (35.2)		38 (33.0)	19 (28.4)	19 (39.6)		34 (31.5)	16 (32.7)	18 (30.5)	
CDM staff training	***(N=102)***	***(N=24)***	***(N=78)***		***(N=99)***	***(N=58)***	***(N=41)***		***(N=94)***	***(N=43)***	***(N=51)***	
Adequate (n, %)	65 (63.7)	17 (70.8)	48 (61.5)	0.56	61 (61.6)	35 (60.3)	26 (63.4)	0.92	59 (62.7)	25 (58.1)	34 (66.7)	0.52
Inadequate (n, %)	37 (36.3)	7 (29.2)	30 (38.5)		38 (38.4)	23 (39.6)	15 (36.6)		35 (37.3)	18 (41.9)	17 (33.3)	
**CDM resource for COPD**												
CDM staff availability	***(N=117)***	***(N=27)***	***(N=90)***		***(N=113)***	***(N=66)***	***(N=47)***		***(N=106)***	***(N=48)***	***(N=58)***	
Adequate (n, %)	79 (67.5)	21 (77.8)	58 (64.4)	0.29	75 (66.4)	47 (71.2)	28 (59.6)	0.27	72 (67.9)	32 (66.7)	40 (69.0)	0.96
Inadequate (n, %)	38 (32.5)	6 (22.2)	32 (35.6)		38 (33.6)	19 (28.8)	19 (40.4)		34 (32.1)	16 (33.3)	18 (31.0)	
CDM staff training	***(N=102)***	***(N=24)***	***(N=78)***		***(N=99)***	***(N=57)***	***(N=42)***		***(N=94)***	***(N=44)***	***(N=50)***	
Adequate (n, %)	63 (61.7)	17 (70.8)	46 (59.0)	0.42	59 (59.6)	33 (57.9)	26 (61.9)	0.84	57 (60.6)	26 (59.0)	31 (62.0)	0.94
Inadequate (n, %)	39 (38.3)	7 (29.2)	32 (41.0)		40 (40.4)	24 (42.1)	16 (38.1)		37 (39.4)	18(41.0)	19 (38.0)	
**CDM resource for IHD**												

CDM staff availability	***(N=119)***	***(N=28)***	***(N=91)***		***(N=115)***	***(N=67)***	***(N=48)***		***(N=108)***	***(N=49)***	***(N=59)***	
Adequate (n, %)	84 (70.6)	22 (78.6)	62 (68.1)	0.41	80 (69.6)	51 (76.1)	29 (60.4)	0.11	77 (71.3)	33(67.3)	44 (74.6)	0.54
Inadequate (n, %)	35 (29.4)	6 (21.4)	29 (31.9)		35 (30.4)	16 (23.9)	19 (39.6)		31 (28.7)	16 (32.7)	15 (25.4)	
CDM staff training	***(N= 101***	***(N=24)***	***(N=77)***		***(N=98)***	***(N=57)***	***(N=41)***		***(N=93)***	***(N=43)***	***(N=50)***	
Adequate (n, %)	69 (68.3)	18 (75.0)	51 (66.2)	0.58	65 (66.3)	38 (66.7)	27 (65.9)	0.929	63 (67.7)	27 (62.8)	36 (72.0)	0.47
Inadequate (n, %)	32 (31.7)	6 (25.0)	26 (33.8)		33 (33.7)	19 (33.3)	14 (34.1)		30 (32.3)	16(37.2)	14 (28.0)	
**CDM resource for Diabetes**												
CDM staff availability	***(N=119)***	***(N=28)***	***(N=91)***		***(N=115)***	***(N=67)***	***(N=48)***		***(N=108)***	***(N=49)***	***(N=59)***	
Adequate (n, %)	87 (73.1)	23 (82.1)	64 (70.3)	0.32	83 (72.2)	53 (79.1)	30 (62.5)	0.08	80 (74.0)	35 (71.4)	45 (76.3)	0.72
Inadequate (n, %)	32 (26.9)	5 (17.9)	27 (29.7)		32 (27.8)	14 (20.9)	18 (37.5)		28 (26.0)	14 (28.6)	14 (23.7)	
CDM staff training	***(N=101)***	***(N=24)***	***(N77=)***		***(N=98)***	***(N=57)***	***(N=41)***		***(N=93)***	***(N=43)***	***(N=50)***	
Adequate (n, %)	73 (72.3)	18 (75)	55 (71.4)	0.94	69 (70.4)	41 (71.9)	28 (68.3)	0.87	67 (72.0)	29 (67.4)	38 (76.0)	0.49
Inadequate (n, %)	28 (27.7)	6 (25)	22 (28.6)		29 (29.6)	16 (28.1)	13 (31.7)		26 (28.0)	14 (32.6)	12 (24.0)	
**Expansion of the other programs to HSE CDM program**												
Mental health	***(N=119)***	***(N=27)***	***(N=92)***		***(N=115)***	***(N=68)***	***(N=47)***		***(N=109)***	***(N=49)***	***(N=60)***	
Agree (n, %)	80 (67.2)	20 (74.1)	60 (65.2)	0.53	78 (67.8)	44 (64.7)	34 (72.3)	0.51	73 (67.0)	37 (75.5)	36 (60.0)	0.13
Disagree (n, %)	39 (32.8)	7 (25.9)	32 (34.8)		37 (32.2)	24 (35.3)	13 (27.7)		36 (33.0)	12 (24.5)	24 (40.0)	
Traveller health	***(N=115)***	***(N=27)***	***(N=88)***		***(N=111)***	***(N=65)***	***(N=46)***		***(N=107)***	***(N=49)***	***(N=58)***	
Agree (n, %)	73 (63.5)	19 (70.4)	54(61.4)	0.53	72 (64.9)	41 (63.1)	31 (67.4)	0.79	67 (62.6)	30 (61.2)	37 (63.8)	0.94
Disagree (n, %)	42 (36.5)	8 (29.6)	34 (38.6)		39 (35.1)	24 (36.9)	15 (32.6)		40 (37.4)	19 (38.8)	21 (36.2)	
Obesity	***(N=121)***	***(N=28)***	***(N=93)***		***(N=117)***	***(N=68)***	***(N=49)***		***(N=111)***	***(N=51)***	***(N=60)***	
Agree (n, %)	102 (84.3)	25 (89.3)	77 (82.8)	0.56	99 (84.6)	58 (85.3)	41 (83.7)	0.80	94 (84.7)	43 (84.3)	51 (85.0)	0.97
Disagree (n, %)	19 (15.7)	3 (10.7)	16 (17.2)		18 (15.4)	10 (14.7)	8 (16.3)		17 (15.3)	8 (15.6)	9 (15.0)	
Osteoporosis	***(N=116)***	***(N=27)***	***(N=89)***		***(N=112)***	***(N=66)***	***(N=46)***		***(N=107)***	***(N=47)***	***(N=60)***	
Agree (n, %)	88 (75.9)	23 (85.2)	65 (73.0)	0.30	85 (75.9)	50 (75.8)	35 (76.0)	0.92	81 (75.7)	36 (76.6)	45 (75.0)	0.94
Disagree (n, %)	28 (24.1)	4 (14.8)	24 (27.0)		27 (24.1)	16 (24.2)	11 (24.0)		26 (24.3)	11 (23.4)	15 (25.0)	

P-values calculated using chi-square test

Supplementary table S4: Nearby local services and satisfaction towards waiting times by practice location, size, and post-graduate training.

Nearby local services	Rural				Post graduate training centres				GMS patients			
	**All**	**Yes**	**No**	**P value**	**All (n, %)**	**Yes**	**No**	**P value**	**All (n, %)**	**<=1500 52**	**>=1500 61**	**P value**
**Cardiac Echo**	**N=111**	**N= 25**	**N=86**		**N=107**	**N=61**	**N=46**		**N=102**	**N=48**	**N=54**	
Availability (n, %)	76 (68.5)	21(84.0)	55 (63.9)	0.09	73 (68.2)	41 (67.2)	32 (69.6)	0.96	71 (69.6)	33 (68.7)	38 (70.4)	0.97
Waiting times	***N=103***	***N=27***	***N=76***		**N=99**	**N=59**	**N=40**		**N=94**	**N=46**	**N=48**	
Adequate (n, %)	31 (30.1)	9 (33.3)	22 (28.9)	0.85	31 (31.3)	20 (33.9)	11 (27.5)	0.65	26 (27.7)	12 (26.1)	14 (29.2)	0.92
Inadequate (n, %)	72 (69.9)	18 (66.7)	54 (71.1)		68 (68.7)	39 (66.1)	29 (72.5)		68 (72.3)	34 (73.9)	34 (70.8)	
**Exercise Stress Test**	**N=110**	**N=25**	**N=85**		**N=106**	**N=61**	**N=45**		**N=101**	**N=48**	**N=53**	
Availability (n, %)	83 (75.4)	20 (80.0)	63 (74.1)	0.74	79 (74.5)	41 (67.2)	38 (84.4)	0.07	75 (74.3)	37 (77.1)	38 (71.2)	0.70
Waiting times	***N=106***	***N=27***	***N=79***		***N=102***	***N=58***	***N=44***		**N=96**	**N=49**	**N=47**	
Adequate (n, %)	21 (19.8)	8 (29.6)	13 (16.4)	0.30	20 (19.6)	14 (24.1)	6 (13.6)	0.28	19 (19.8)	8 (16.3)	11 (23.4)	0.54
Inadequate (n, %)	85 (80.2)	19 (70.4)	66 (83.6)		82 (80.4)	44 (75.9)	38 (86.4)		77 (80.2)	41 (83.7)	36 (76.6)	
**Physiotherapy**	**N=113**	**N=25**	**N=88**		**N=109**	**N=62**	**N=47**		**N=104**	**N=49**	**N=55**	
Availability (n, %)	112 (99.1)	25 (100)	87 (98.9)	0.96	108 (99.1)	61 (98.4)	47 (100)	0.92	103 (99.1)	48 (97.9)	55 (100)	0.47
Waiting times	***N=119***	***N=27***	***N=92***		***N=115***	***N=67***	***N=48***		**N=109**	**N=51**	**N=58**	
Adequate (n, %)	31 (26.1)	11 (40.7)	20 (21.7)	0.08	30 (26.1)	19 (28.4)	11 (22.9)	0.66	29 (26.6)	12 (23.5)	17 (29.3)	0.64
Inadequate (n, %)	88 (73.9)	16 (59.3)	72 (78.3)		85 (73.9)	48 (71.6)	37 (77.1))		80 (73.4)	39 (76.5)	41 (70.7)	
**Dietician**	**N=113**	**N=25**	**N=88**		**N=109**	**N=62**	**N=47**		**N=104**	**N=49**	**N=55**	
Availability (n, %)	100 (88.5)	25 (100)	75 (85.3)	0.07	96 (88.1)	53 (48.6)	43 (39.4)	0.39	91 (87.5)	45 (91.8)	46 (83.6)	0.25
Waiting times	***N=113***	***N=27***	***N=86***		***N=109***	***N=62***	***N=47***		**N=103**	**N=50**	**N=53**	
Adequate (n, %)	28 (24.8)	8 (29.6)	20 (23.3)	0.68	27 (24.8)	15 (24.2)	12 (25.5)	0.92	25 (24.3)	13 (26.0)	12 (22.6)	0.87
Inadequate (n, %)	85 (75.2)	19 (70.4)	66 (76.7)		82 (75.2)	47 (75.8)	35 (74.5)		78 (75.7)	37 (74.0)	41 (77.4)	
**Retinal screening**	**N=111**	**N=25**	**N=86**		**N=107**	**N=61**	**N=46**		**N=102**	**N=48**	**N=54**	
Availability (n, %)	110 (99.1)	24 (96.0)	86 (100)	0.51	106 (99.1)	60 (98.4)	46 (100)	0.92	101 (99.1)	47 (97.9)	54 (100)	0.47
Waiting times	***N=118***	***N=27***	***N=91***		***N=114***	***N=66***	***N=48***		**N=108**	**N=51**	**N=57**	
Adequate (n, %)	93 (78.8)	20 (74.1)	73 (80.2)	0.67	91 (79.8)	54 (81.8)	37 (77.1)	0.70	88 (81.5)	40 (78.4)	48 (84.2)	0.60
Inadequate (n, %)	25 (21.2)	7 (25.9)	18 (19.8)		23 (20.2)	12 (18.2)	11 (22.9)		20 (18.5)	11 (21.6)	9 (15.8)	
**Smoking cessation**	**N=109**	**N=25**	**N=84**		**N=105**	**N=59**	**N=46**		**N=100**	**N=47**	**N=53**	
Availability (n, %)	99 (90.8)	22 (88.0)	77 (91.7)	0.69	96 (91.4)	53 (89.8)	43 (93.5)	0.73	92 (100)	43 (91.5)	49 (92.5)	0.91
Waiting times	***N=111***	***N=25***	***N=86***		***N=108***	***N=61***	***N=47***		**N=103**	**N=47**	**N=54**	
Adequate (n, %)	59 (53.2)	14 (56.0)	45 (52.3)	0.92	58 (53.7)	32 (52.5)	26 (55.3)	0.92	57 (55.3)	27 (55.1)	30 (55.6)	0.98
Inadequate (n, %)	52 (46.8)	11 (44.0)	41 (47.7)		50 (46.3)	29 (47.5)	21 (44.7)		46 (44.7)	22 (44.9)	24 (44.4)	

P-values calculated using chi-square test

Supplementary figure S1: Overall staffing by practice location, size, and post-graduate training.

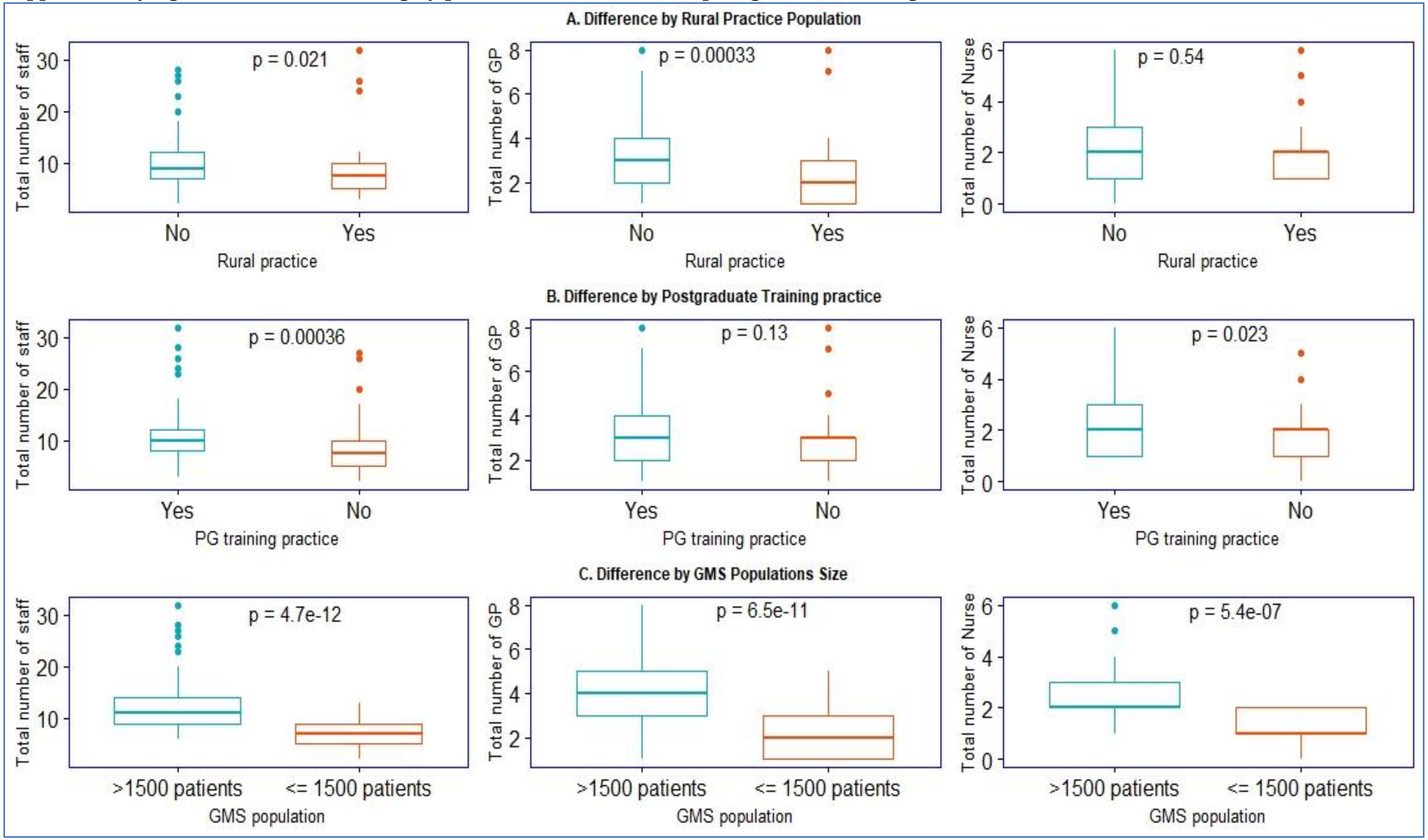

Note: ***p-value significant at P<0.05.**
